# Metabolic engineering of *Shewanella oneidensis* to produce glutamate and itaconic acid

**DOI:** 10.1007/s00253-023-12879-5

**Published:** 2024-01-06

**Authors:** Hannah Wohlers, Laura Zentgraf, Lisa van der Sande, Dirk Holtmann

**Affiliations:** 1 Institute of Bioprocess Engineering and Pharmaceutical Technology, University of Applied Sciences Mittelhessen, Wiesenstrasse 14, 35390 Giessen, Germany; 2 DECHEMA-Forschungsinstitut, Microbial Biotechnology, Theodor-Heuss-Allee 25, 60486 Frankfurt Am Main, Germany; 3 Institute of Process Engineering in Life Sciences, Karlsruhe Institute of Technology, Karlsruhe, Fritz-Haber-Weg 4, 76131 Karlsruhe, Germany

**Keywords:** *Shewanella oneidensis*, Glutamate, Itaconic acid, Metabolic engineering

## Abstract

**Abstract:**

*Shewanella oneidensis* is a gram-negative bacterium known for its unique respiratory capabilities, which allow it to utilize a wide range of electron acceptors, including solid substrates such as electrodes. For a future combination of chemical production and electro-fermentation, the goal of this study was to expand its product spectrum. *S. oneidensis* was metabolically engineered to optimize its glutamate production and to enable production of itaconic acid. By deleting the glutamate importer *gltS* for a reduced glutamate uptake and *pckA/ptA* to redirect the carbon flux towards the TCA cycle, a ∆3 mutant was created. In combination with the plasmid pG2 carrying the glutamate dehydrogenase *gdhA* and a specific glutamate exporter *NCgl1221 A111V*, a 72-fold increase in glutamate concentration compared to the wild type was achieved. Along with overexpression of *gdhA* and *NCgl1221 A111V*, the deletion of *gltS* and *pckA/ptA* as well as the deletion of all three genes (∆3) was examined for their impact on growth and lactate consumption. This showed that the redirection of the carbon flux towards the TCA cycle is possible. Furthermore, we were able to produce itaconic acid for the first time with a *S. oneidensis* strain. A titer of 7 mM was achieved after 48 h. This suggests that genetic optimization with an expression vector carrying a cis-aconitate decarboxylase (*cadA*) and a aconitate hydratase (*acnB*) along with the proven redirection of the carbon flux to the TCA cycle enabled the production of itaconic acid, a valuable platform chemical used in the production of a variety of products.

**Key points:**

*•Heterologous expression of gdhA and NCgl1221_A111V leads to higher glutamate production.*

*•Deletion of ackA/pta redirects carbon flux towards TCA cycle.*

*•Heterologous expression of cadA and acnB enables itaconic acid production.*

**Supplementary Information:**

The online version contains supplementary material available at 10.1007/s00253-023-12879-5.

## Introduction

### Glutamate and itaconic acid

Natural amino acids like glutamate have a wide range of uses in the chemical and biotechnological industries. The most prevalent kind of glutamate in the food business is monosodium glutamate. In the pharmaceutical sector, glutamate is used to produce a variety of drugs, including antibiotics, anticancer drugs, and immunosuppressants (Ault 2004). The glutamic acid market is predicted to be valued USD 9.54 billion in 2020 and is expected to increase at a compound yearly growth rate of 7.6% from 2021 to 2028, reaching USD 17.16 billion. With a revenue share of more than 80% in 2020, the food and beverage application category led the glutamic acid market (Market Analysis Report 2022). Glutamate is mainly manufactured through microbial fermentation. Kinoshita et al. (2004) discovered the L-glutamate-producing bacterium, *Corynebacterium glutamicum*, originally designated *Micrococcus glutamicus* in 1957. Several bacteria applicable for glutamate production have been isolated since (Sano 2009). In general, commercially effective producers have been created by gradually accumulating advantageous genetic and phenotypic traits in a single background using traditional mutagenesis and/or recombinant DNA technologies. Making use of those technologies, a combination of gene deletion and plasmid based heterologous expression seemed applicable. In this study, the aim was to redirect the carbon flux from acetate towards the TCA cycle for an enhanced glutamate production and to reduce the reuptake of glutamate by host cells. Therefore, genes like *pckA/ptA* of *Shewanella oneidensis* MR-1 coding for enzymes catalyzing the ATP-generating steps from acetyl-CoA to acetate (Hunt et al. 2010) were targeted for deletion as well as a glutamate importer (*gltS*) to reduce the reuptake of secreted glutamate. To further optimize the glutamate production, an inducible vector pJem1 (obtained from (Jeske and Altenbuchner 2010) was constructed with the glutamate dehydrogenase *gdhA* and the specific glutamate exporter *NCgl1221 A111V* from *Corynebacterium glutamicum* (Nakamura et al. 2007). Such advances entail strains like *S. oneidensis* that are able to produce glutamate at better yields as well as fewer byproducts, as their removal accounts for the majority of expenses during downstream processing (Zhang et al. 2014; Kogure and Inui 2018).

Itaconic acid (IA) is a flexible platform substance that may be utilized as a monomer to create a variety of polymers, including biodegradable plastics (Sriariyanun et al. 2019; Ray et al. 2017). Asia Pacific accounts for about 54% of the worldwide demand for itaconic acid, with Europe and North America coming in second and third. Methyl methacrylate, polyitaconic acid, and SBR (styrene-butadiene rubber) latex manufacture account for a sizeable percentage of the itaconic acid market. The global market was worth around $75 million in 2015, growing to $95.4 million by 2021, and is predicted to be worth around $110.4 million by 2028 (Global Market Insights 2016; Market Data Forecast 2023). *Aspergillus itaconicus* was shown by (Kinoshita 1932) to create IA when grown on D-glucose, providing the first instance of a microbe capable of doing so. The Northern Regional Research Laboratory (NRRL) of the United States Department of Agriculture discovered *Aspergillus terreus* as a unique producer of IA in 1939 and identified *A. terreus* NRRL 1960 as a strain able to generate large titers of IA (80 g L^−1^) (Lockwood and Nelson 1946). Because of *A. terreus*’ extreme sensitivity to contaminants (e.g., manganese) in the fermentation broth (Karaffa et al. 2015) and the morphological diversity (in particular as pellets of different sizes or filamentous) in which this fungus can grow (Krull et al. 2017), its utilization for IA synthesis in an industrial context is challenging, pointing out the need for new production hosts. This study approaches to modify *S. oneidensis* genetically to produce IA from its naturally produced intermediate cis-aconitate. The same steps as for the enhanced glutamate production were taken here to redirect the carbon flux towards the TCA cycle. The missing enzyme for IA production, a cis-aconitate decarboxylase from *A. terreus* itself (*cadA*), will be heterologously expressed on the inducible vector pJem1 (obtained from (Jeske and Altenbuchner 2010). To increase the production of the precursor cis-aconitate and consequently IA production, it would be reasonable to overexpress *S. oneidensis acnB*, which encodes the aconitate hydratase.

### Shewanella oneidensis and bioelectrotechnology

*S. oneidensis* is a gram-negative bacterium known for its unique respiratory capabilities that allow it to utilize a variety of electron acceptors, including solid substrates such as electrodes (Marsili et al. 2008). Due to this characteristic, *S. oneidensis* has become a popular choice for the realization of microbial electrosynthesis (MES) and microbial fuel cells (MFC), which depend on the transmission of electrons from microorganisms to an electrode to produce energy or chemicals (Ikeda et al. 2021). Schröder et al. (2015) define MES as “the execution of microbially catalyzed electrochemical reactions to transform a substance into a desired product.” Electroactive bacteria (EAB) can transfer electrons across biological membranes to connect intracellular and external electron acceptor/donor systems. These EAB may be utilized in either MFC or MES. Bacteria in MFCs oxidize organic fuels such as lactate as well as complex combinations of organic matter and transfer the biologically produced electrons to the anode, resulting in current generation. Depending on the intended reaction, MES can be classified as cathodic or anodic. Bacteria in cathodic MES receive electrons given by external sources (e.g., electrodes) and utilize them in reduction processes (e.g., reductive carbon fixation (Rabaey et al. 2009)). In anodic MES, bacteria give off electrons during an unbalanced fermentation (Flynn et al. 2010). MES applications for *S. oneidensis* include, for instance, bioremediation of dyes (Gomaa et al. 2017; Li et al. 2018b), metal ions (Han et al. 2017), nitrobenzene (Wang et al. 2013), and sulfonamides (Mao et al. 2018).

Extracellular electron transfer is characterized in three ways: direct electron transfer (DET), mediated electron transfer (MET), and indirect electron transfer (IET). DET necessitates physical contact with the electrode, which may be achieved by the use of pili, nanowires, or membrane-bound cytochromes. It is commonly used by bacteria that create biofilms, such as *Geobacter sulfurreducens*. No physical contact is required for the MET; instead, bacteria such as *S. oneidensis* employ redox active mediator molecules such as flavines to transport electrons between the cell and the electrode. These mediator molecules can be regenerated electrophilically unlike electron-shuttling chemicals needed for IET like hydrogen or formic acid which are consumed by microorganisms such as *C. necator* (Sydow et al. 2014)*.*

The aim of this study was to broaden the product spectrum of *S. oneidensis* by metabolic engineering for a future combination of chemical production with electro-fermentation. So far, the production of isobutanol (Jeon et al. 2015), acetoin (Bursac et al. 2017), and formic acid (Le Tuan et al. 2018) could be shown with *S. oneidensis*.

## Materials and methods

### Chemicals

All chemicals were obtained from Carl Roth (Karlsruhe, Germany), VWR International (Darmstadt, Germany), or Merck (Darmstadt, Germany). Solvents for chromatography were acquired of LC–MS grade quality.

### Bacterial strains and growth conditions

*Escherichia coli* DH5α (ATCC 67879 (Hanahan 1985; Grant et al. 1990)) and *E. coli* WM3064 cultures were grown in Lysogeny broth (LB) medium (Bertani 1951) at 37 °C. *E. coli* WM3064 cultures were supplemented with 0.3 mM of 2,6-diaminopimelic acid (DAP) and supplied with 50 μg/mL of kanamycin sulfate when necessary. For cultivation of *S. oneidensis* MR-1 (ATCC 700550 (Myers and Nealson 1988)), lactate *Shewanella* basal medium (LSBM) was prepared containing the following compounds per liter: KH_2_PO_4_ 225 mg, K_2_HPO_4_ 225 mg, NaCl 460 mg, (NH_4_)_2_SO_4_ 225 mg, MgSO_4_ × 7 H_2_O 117 mg, HEPES 23.8 g, lactate (90% solution) 22.4 g (100 mM), 5 mL vitamin solution, and 5 mL trace mineral solution. The pH was adjusted to 7.2 before adding the vitamin and trace mineral solution using a 1 M NaOH solution made from NaOH pellets. The vitamin solution contained per liter biotin 2 mg, folic acid 2 mg, pyridoxine hydrochloride 10 mg, thiamin hydrochloride 5 mg, riboflavin 5 mg, nicotinic acid 5 mg, DL-calcium pantothenate 5 mg, cyanocobalamine 0.1 mg, 4-aminobenzoic acid 5 mg, and lipoic acid 5 mg. The trace mineral solution contained the following ingredients per liter: nitrilotriacetic acid (C_6_H_9_NO_6_) 1.5 g, MgSO_4_ × 7 H_2_O 3 g, MnSO_4_ × 2 H_2_O 0.5 g, NaCl 1 g, FeSO_4_ × 7 H_2_O 100 mg, CoCl_2_ 100 g, CaCl_2_ × 2 H_2_O 100 mg, ZnCl_2_ 130 mg, CuSO_4_ × 5 H_2_O 10 mg, AlK(SO_4_)_2_ 10 mg, H_3_BO_3_ 10 mg, Na_2_MoO_4_ × 2 H_2_O 25 mg, NiCl_2_ 24 mg, and Na_2_WO_4_ × 2 H_2_O 25 mg. The LSBM main solution was sterilized for 20 min at 121 °C, and the vitamin and trace mineral solutions were sterile filtered and kept at 4 °C until further use. Five milliliter precultures in LB medium were cultured in test tubes for 24 h at 30 °C and 180 rpm in an orbital shaking incubator (Minitron HT, Infors, Bottmingen, Switzerland) for growth of *S. oneidensis* MR-1 in liquid media. Main cultures of 25 mL in LSBM supplemented with 0.1% (wt/vol) casein hydrolysate were subsequently inoculated to an OD_600_ of 0.05 in 100 mL shake flasks and incubated under the same conditions. If required, kanamycin sulfate at 50 μg/mL was added to the medium. For promoter induction from plasmid pJeM1 and pG2, l-rhamnose was added to a final concentration of 0.2% (wt/vol) at OD_600_ of 0.15–0.3 in liquid medium if not stated otherwise.

### Cultivation of S. oneidensis MR-1 in BioLector microbioreactor and fluorescence assay

The precultures of *S. oneidensis* MR-1 were grown in LB for 16 h and used for the inoculation of 1 mL medium at the starting OD_600_ of 0.05. The cultivation was carried out in a BioLector® MB system (m2p-labs, Germany) in MTP-48 FlowerPlates® with pH optodes at 30 °C, 1000 rpm, and 95% humidity. The growth of the cultures was tracked online using scattered light signal monitoring. To examine the induction of the *rhaBAD* promoter by different concentrations of l-rhamnose and its influence on bacterial growth, fluorescence of bacterial suspension as well as the optical density was continuously recorded. The fluorescence intensity at 488 nm excitation wavelength and 520 nm emission wavelength was measured and normalized by the optical density at 600 nm.

### DNA cloning and plasmid construction

All plasmid cloning techniques were carried out in *E. coli* DH5α. Thermo Scientific’s GeneJET Plasmid Miniprep Kit (Waltham, USA) was used to purify plasmid DNA. Polymerase chain reactions (PCR) were carried out according to the manufacturer’s procedure using Q5 Polymerase from New England Biolabs (Frankfurt, Germany). Following that, PCR products were purified using the Zymo Research Europe (Freiburg, Germany) DNA Clean & Concentrator Kit. Vectors were assembled using the isothermal in vitro recombination (Gibson et al. 2009). Oligonucleotides were purchased from Merck (Darmstadt, Germany) and restriction enzymes and T4 ligase from NEB. Sanger sequencing at Eurofins Scientific (Luxembourg, Luxembourg) verified all genetic constructs. Transformation of *E. coli* DH5α and WM3064 was done via electroporation. Vectors were conjugated in *S. oneidensis* MR-1 with *E. coli* WM3064 as donor strain. Genomic DNA of *S. oneidensis* MR-1 was purified with GenElute™ Bacterial Genomic DNA Kit from Merck (Darmstadt, Germany). Oligonucleotides, plasmids, and strains used in this work can be found in Table S1 in the supplementary information.

### Generation of deletion mutants

Knockout of the genes *ackA* (SO 2915), *pta* (SO 2916), and *gltS* (SO3562) was carried out with allelic exchange vector pNTPS138-R6KT as described by Lassak et al. (2010). Upstream and downstream portions (approximately 500 bp) of the targeted gene area, as well as the backbone vector pNTPS138-R6KT, were amplified by PCR using the relevant primer pairs (given in Table S1 in the supplementary information). Gibson Assembly fused the fragments after purification.

### Metabolite analysis

Metabolites were quantified by HPLC equipped with a UV–visible-light (Vis) detector and refractive index detector. The HPLC system consisted of an SPD-20A UV–Vis detector, two LC-20AT pumps, a SIL-20AC autosampler, a CTO-20AC column oven, and an RID-10A detector. The units used are from the manufacturer Shimadzu Deutschland GmbH (Duisburg, Germany). A Rezex™ ROA-Organic Acid H + 8% column (250 × 4.6 mm; Phenomenex, California, USA) with precolumn was used for the analysis. Flow rate was 0.6 mL/min, the column oven temperature 30 °C, injection volume 10 μL, and the separation time was 35 min. The mobile phase consisted of acidified water (0.005 M H_2_SO_4_). For quantification of metabolites, the supernatant of *S. oneidensis* cultures was centrifuged for 5 min at 16.000 g and passed through a 0.22 μm PVDF-syringe filter (Carl Roth, Karlsruhe, Germany). Samples were analyzed at 254 nm for lactate and 202 nm for itaconic acid and acetate. Retention times of the analytes are listed in Table S2 in the supplemental material. For the quantification of glutamate, a LC–MS/MS setup was used. One microliter of each sample was chromatographically purified on a 150 × 4.6 mm Luna Omega 3 m PS C18 100 column (Phenomenex, Aschaffenburg, Germany) in a Nexera X2 UHPLC system (Shimadzu, Duisburg, Germany). The mobile phase consisted of 0.2% formic acid in water (A) and 0.2% formic acid in acetonitrile (B). The pump gradient started at 10% mobile phase B; from 0.10 min, the proportion of mobile phase B was continuously increased linearly until it reached 100% B at 2 min; from 2.50 min to 4.50 min, the proportion of mobile phase B was again decreased linearly to the initial value of 10%. The total run time was 5 min. The flow rate was 0.5 mL/min. The analytes were ionized negatively with an APCI ion source, fragmented, and ultimately quantified by comparing the findings to calibration curves of comparable standards. Shimadzu’s LabSolutions software was used for quantification.

### Software

Plasmid design was performed using SnapGene® software (from Dotmatics; available at snapgene.com). All graphs were generated using Origin(Pro), version 2022b (OriginLab Corporation, Northampton, MA, USA).

### *GenBank accession numbers (*Table 1*)*

**Table 1:** Genes used in this study with corresponding GenBank accession numbers

Gene	Range	GenBank accession
*ackA*	3,045,853 to 3,047,052	AE014299.2
*gltS*	3,054,833 to 3,056,056	AE014299.2
*pta*	3,047,174 to 3,049,327	AE014299.2
*cadA*	9 to 1474	MH366503.1
*acnB*	455,782 to 458,378	AE014299.2
*NCgl1221*	2,117,239 to 2,118,840	CP025534.1
*gdhA*	3,010,764 to 3,012,107	CP025534.1

## Results

### Heterologous expression of gdhA and NCgl1221

**Fig. 1:**
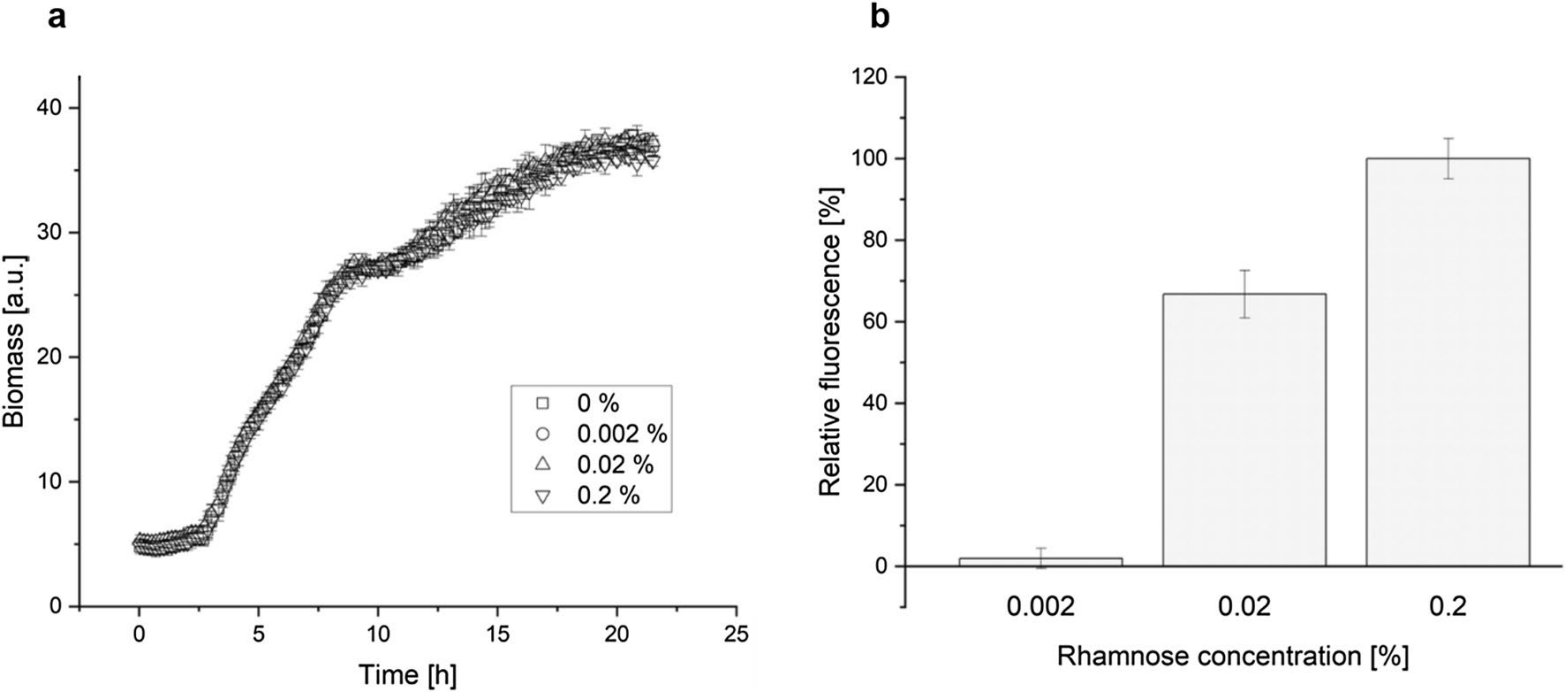
Influence of different L-rhamnose concentrations on **a** *S. oneidensis’* growth and **b** relative fluorescence (0.2% rhamnose was set as 100%). The cultivations and measurements were performed in BioLector microbioreactor; the data points are the averages and standard deviations of three biological replicates. GFP fluorescence was measured at 520 nm

*S. oneidensis* can use fermentation end products like lactate, formate, and hydrogen as electron donors and is capable of using a broad range of electron acceptors (e.g., fumarate, metal ions). This makes it an attractive host for biotechnological applications. The production of glutamate was studied as a model reaction to see if it is possible to modify *S. oneidensis’* citrate cycle for the production of bulk chemicals. For this, the genes from *Corynebacterium glutamicum* for a glutamate dehydrogenase *gdhA* as well as the mutant of a mechano-selective glutamate exporter *NCgl1221 A111V* (Nakamura et al. 2007) were expressed from a pJeM1 plasmid (obtained from (Jeske and Altenbuchner 2010)) under control of the *rhaBAD* operon. As there was no published data about the use of this operon in *S. oneidensis* at the time of these experiments, its expression strength was measured using pJeM1 with GFP (green fluorescent protein) as reporter. Different rhamnose concentrations (0.002, 0.02, and 0.2%) were tested to observe the inducer’s impact on *S. oneidensis’* growth (Fig. 1A) and to assess the promoter’s regulation measured via fluorescence (Fig. 1B). Later, Cheng and colleagues engineered a rhamnose-inducible system for various *Shewanella* species, which confirms our results (Cheng et al. 2021).

Higher concentrations of rhamnose led to an increase in relative fluorescence and had no impact on *S. oneidensis* growth. As this shows that the *rhaBAD* operon is suitable for *S. oneidensis*, the following experiments were conducted with the above-mentioned plasmid system, further referred to as pG2. The glutamate production of the wild-type strain carrying pG2 was compared to genetically optimized strains later on (Fig. 4).

### Optimization of glutamate production by gene deletions

**Fig. 2:**
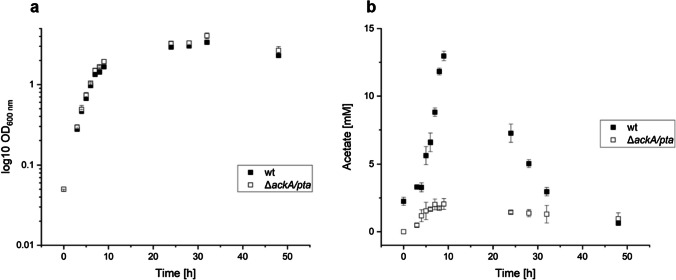
Influence of *ackA/pta* deletion on **a** *S. oneidensis’* growth and **b** its acetate production. The data points are the averages and standard deviations of three biological replicates

In addition to overexpression, several gene deletions were screened for their influence on glutamate production. *S. oneidensis* metabolizes lactate via pyruvate and acetyl-CoA to acetate using lactate as an electron sink if the amount of electron acceptor is limited (Takuya Kasai et al. 2019). The *ackA/pta* genes, which catalyze the ATP-generating steps from acetyl-CoA to acetate, were deleted to reroute the carbon flux from acetate to the TCA cycle (Hunt et al. 2010). *S. oneidensis’* growth under aerobic conditions was not impacted by the deletions and is comparable to the wild type (Fig. 2a). In the deletion mutant ∆*ackaA*∆*pta* (∆2), generated with the pNPTS138-R6KT_ackApta deletion plasmid, acetate production (Fig. 2b) decreased significantly and remained at around 2 mM, probably due to background activity, while acetate production reached around 14 mM in the wild type after 9 h. After 24 h, a decrease in acetate concentration is observed in the wild type as it is taken up and metabolized again.

For further optimization of the glutamate production, reuptake of the produced glutamate must be prevented. Therefore, the gene *gltS* which codes for a glutamate transporter was deleted with the deletion plasmid pNPTS138-R6KT_gltS.

**Fig. 3:**
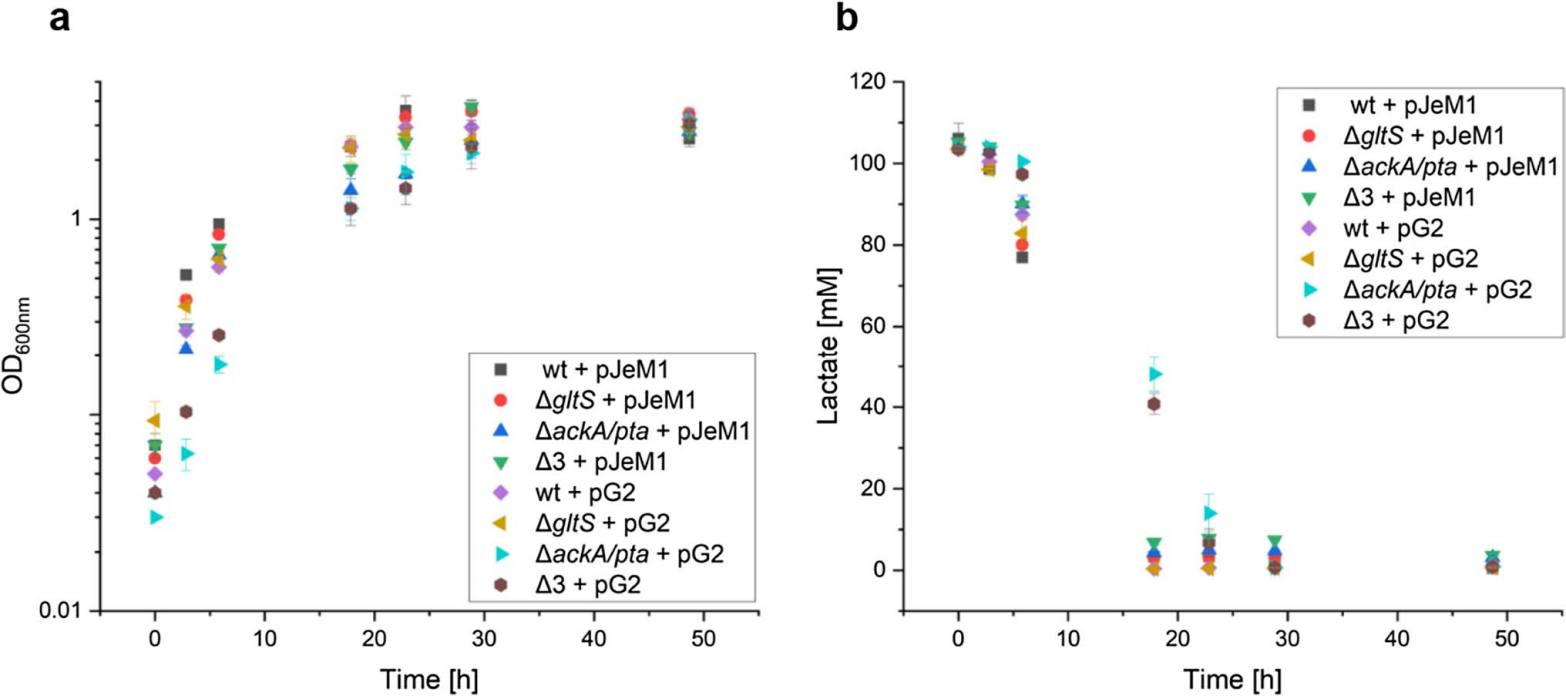
Influence of gene deletions and plasmids on **a** *S. oneidensis’* growth and **b** lactate consumption. Δ3: mutant with deletion of *ackA/pta* and *gltS*; pJeM1: empty plasmid; pG2: plasmid containing glutamate production genes. The data points are the averages and standard deviations of three biological replicates

The wild-type and deletion mutants were grown with the empty plasmid pJeM1 as well as the generated plasmid pG2 to determine the gene deletion’s and plasmid’s influence on growth and substrate consumption. Under aerobic conditions, neither appeared to affect *S. oneidensis’* growth (Fig. 3a) or lactate consumption (Fig. 3b). The slight differences in OD_600nm_ are due to slightly varying initial values, but the growth curves follow the same pattern.

**Fig. 4:**
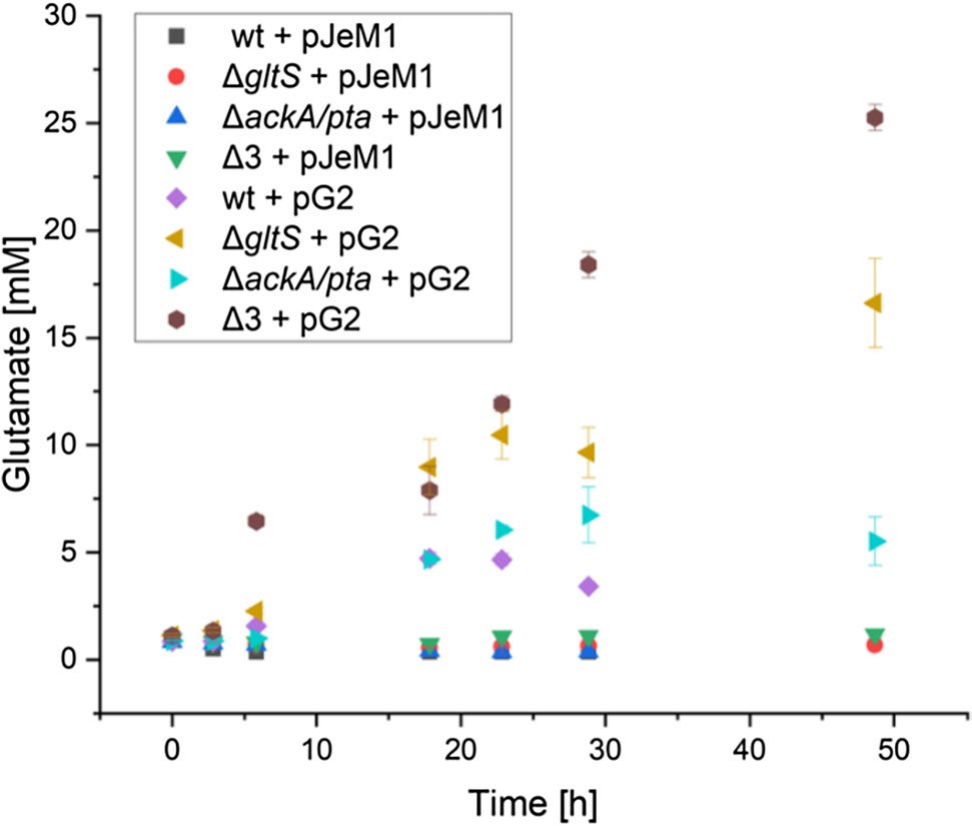
Influence of gene deletions and plasmids on glutamate production. Δ3: mutant with deletion of *ackA/pta* and *gltS*; pJeM1: empty plasmid; pG2: plasmid containing glutamate production genes. The data points are the averages and standard deviations of three biological replicates

Comparing the influence of the empty plasmid (pJeM1) with plasmid pG2, a significantly higher glutamate production was evident for the strains with the latter, even though no clear overproduction of the proteins GdhA and NCgl1221 was evident on the SDS PAGE gels (data not shown). The glutamate concentrations in the strains carrying the empty plasmid remained below 1 mM (Fig. 4.).

The Δ3 deletion mutant showed the highest glutamate concentration with 25 mM, while that of the Δ*gltS* mutant was just under 17 mM. The glutamate concentration of the Δ*ackApta* mutant was approximately 7 mM, and that of the wild type reached just under 5 mM, decreasing again after 22 h. It seems that the deletion of the glutamate exporter has the highest impact on the glutamate concentration.

Since one glutamate molecule is produced from three lactate molecules (Eq. 1), it can be deduced that with the Δ3 deletion mutant nearly 76% of the theoretical maximum of the consumed lactate was converted into glutamate. Considering the differences in fermentation, especially the lower biomass for *S. oneidensis*, the biomass-specific glutamate production is in a comparable range to that of industrially used *C. glutamicum* strains (Table 2). The final product concentrations in the newly developed system are even lower by a factor of 27. Therefore, it can be concluded that the next step here is to optimize the growth of *S. oneidensis*.

**Table 2:** Comparison of glutamate-producing strains (Ault 2004; Abdenacer et al. 2021; Zhang et al. 2014)

Strain	Glutamic acid titer (g/L)	Glutamic acid yield *Y*_P/S_^a^ (%)	Biomass (g/L)	Carbon source Fermentation	Reference
*C. glutamicum*	100.00	60.0	Not shown	Carbohydrates + sugars Batch	(Ault 2004)
*C. glutamicum* 2262	58.57	66.6	18.52	Sugars from date juice Batch	(Abdenacer et al. 2021)
*C. glutamicum* GDK-9	64.5 ± 1.33	–	9.97	Glucose Fed-batch	(Zhang et al. 2014)
*S. oneidensis* MR-1 pG2	0.7 ± 0.1	7.7 ± 0.5 (4.7 ± 0.3^b,c^)	1.33 ± 0.03	Lactate Batch	This work
Δ*gltS* pG2	2.4 ± 0.3	27.3 ± 3.4 (16.7 ± 2.1^b,c^)	1.69 ± 0.20		
Δ*ackA/pta* pG2	1.0 ± 0.2	10.9 ± 2.1 (6.7 ± 1.3^b,c^)	1.24 ± 0.14		
Δ3 pG2	3.7 ± 0.1	41.3 ± 1.0 (25.3 ± 0.6^b,c^)	1.75 ± 0.24		

^a^*Y*_P/S_ = ratio of the produced product and the consumed substrate in g/g

^b^*Y*_P/S_ = ratio of the produced product and the consumed substrate in mol/mol

^c^33% is the theoretical maximum yield with lactate as substrate (Eq. 1)

1 $$3 {{\text{C}}}_{3}{{\text{H}}}_{5}{{\text{O}}}_{3}^{ -} +3\mathrm{ MQ }+ 3 {{\text{NAD}}}^{+}+ 1 {{\text{H}}}_{2}\mathrm{O }+ 1\mathrm{ N}{{\text{H}}}_{4}^{ +}+\left(1 {{\text{C}}}_{4}{{\text{H}}}_{2}{{\text{O}}}_{5}^{ 2-}\right)1 {{\text{C}}}_{5}{{\text{H}}}_{8}{{\text{NO}}}_{4}^{ -}- + 3\mathrm{ MQ}{{\text{H}}}_{2} + 1\mathrm{ NADH }+ 2 (\mathrm{NADH }+ {{\text{H}}}^{+}) + 4\mathrm{ C}{{\text{O}}}_{2} + (1 {{\text{C}}}_{4}{{\text{H}}}_{4}{{\text{O}}}_{3}^{ -} -({\text{S}}-{\text{CoA}}))$$

### Production of itaconic acid

As the metabolic engineering to increase glutamate production was successful, the next step was to broaden the product spectrum of *S. oneidensis.* In order to produce itaconic acid from cis-aconitate, which is a natural intermediate in the citrate cycle of *S. oneidensis*, the gene for a cis-aconitate decarboxylase (*cadA*) from *Aspergillus terreus* must be heterologously expressed. In addition, *S. oneidensis*’ gene for the aconitate hydratase (*acnB*) needs to be overexpressed to achieve a higher production of the precursor cis-aconitate.

**Fig. 5:**
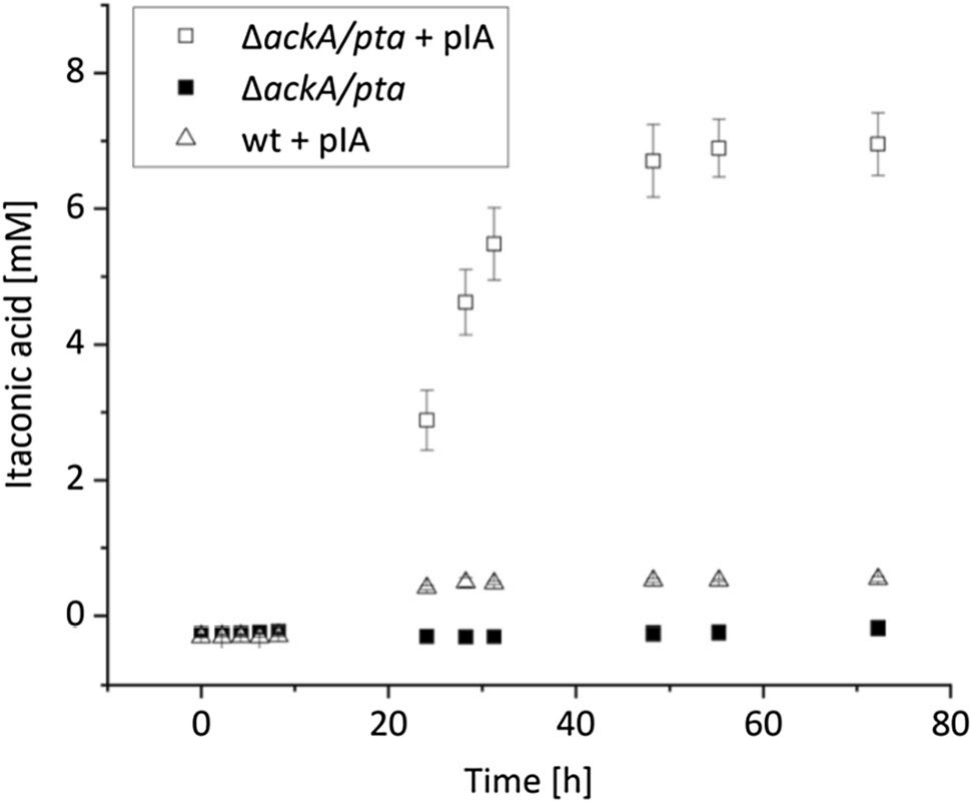
Influence of gene deletions and plasmid-insertion on itaconic acid production. pIA: plasmid containing itaconic acid production genes. The data points are the averages and standard deviations of three biological replicates

According to the glutamate production, the above-mentioned genes (*cadA* and *acnB*) were expressed from a pJeM1 plasmid (obtained from (Jeske and Altenbuchner 2010)) under control of the *rhaBAD* operon, further referred to as plasmid pIA. For the wild type carrying the pIA plasmid, the itaconic acid production remained at around 0.5 mM after 72 h, while the ∆*ackA/pta* mutant with pIA reached titers of nearly 7 mM after 48 h (Fig. 5). Without the plasmid pIA, no itaconic acid production was observed.

Even though the product titers and yields for itaconic acid are low compared to the industrial used strains like *A. terreus* DSM 23081, this is the first reported *S. oneidensis* strain producing itaconic acid (Table 3).

**Table 3:** Comparison of itaconic acid-producing strains (Kuenz et al. 2012; Harder et al. 2018)

Strain	Itaconic acid titer (g/L)	Itaconic acid yield *Y*_P/S_^a^ (%)	Biomass (g/L)	Carbon source Fermentation	Reference
*A. terreus* DSM 23081	65.0	54.2	8.4	Glucose Batch	(Kuenz et al. 2012)
*E. coli* MG1655	46.9 ± 0.5	62.0	6.7	Glucose Fed-batch	(Harder et al. 2018)
*S. oneidensis* MR-1 pIA	0.07 ± 0.01	0.54 ± 0.04 (0.78 ± 0.06)^b^	2.7 ± 0.1	Lactate Batch	This work
Δ*ackA/pta* pIA	0.90 ± 0.06	6.95 ± 0.46 (10.04 ± 0.66)^b^	2.5 ± 0.2		

^a^*Y*_P/S_ = ratio of the produced product and the consumed substrate in g/g

^b^*Y*_P/S_ = ratio of the produced product and the consumed substrate in mol/mol

## Discussion

Metabolic engineering is widely used to develop strains with enhanced abilities for a variety of applications. As *S. oneidensis* MR-1 is a model electroactive bacterium for MFCs, most studies focus on engineering strategies for the optimization of EET (Li et al. 2022, 2018a), broadening the substrate spectrum (Li et al. 2017; Sekar et al. 2016; Choi et al. 2014), and biofilm formation (Mukherjee et al. 2020). In this work, we focused on expanding the product spectrum by redirecting the carbon flux of the TCA cycle. Due to its electroactive characteristics and its genetic accessibility, *S. oneidensis* is a promising host for the coupling of bulk chemical production with MES and MFC applications. As proof of concept, we optimized its glutamate production and successfully achieved a 72-fold increase in glutamate concentration with our deletion mutant ∆3 carrying the plasmid pG2 compared to the wild type. The overexpression of a glutamate dehydrogenase and the glutamate exporter had the highest impact with a 13-fold rise of glutamate, due to the increased abundance of the enzymes that are involved in glutamate synthesis and export. The deletion of the glutamate importer (∆*gltS*) led to a fivefold increase by limiting the reuptake of glutamate and complete secretion into the medium. This emphasizes the power of overexpression of the glutamate dehydrogenase and exporter as only by the blocked reuptake of glutamate the enormous increase of glutamate in the medium is measurable. Another 1.5-fold surge was achieved by deleting the acetate-producing genes as its production no longer competed with the carbon source. To our knowledge, this is the first optimized *Shewanella* strain to produce glutamate at this scale. Furthermore, in this work, we were able to produce itaconic acid for the first time with a *S. oneidensis* strain. Deletion of the acetate-producing genes led to an almost 13-fold increase in itaconic acid production. To further optimize its production via metabolic engineering, the *mfsA* gene, which encodes a membrane permease responsible for the secretion of the produced itaconic acid into the extracellular environment, could also be overexpressed (Huang et al. 2014). Other options include deletion of the malate dehydrogenase gene and downregulation of isocitrate dehydrogenase as shown by (Harder et al. 2016) for *E. coli*.

Glutamate and itaconic acid production both lead to the production of excess electrons and thus to unwanted by-products to maintain the redox balance. Electroactive bacteria, in turn, can transfer these excess electrons to an external electrode, removing the need to produce undesirable side products and enabling stoichiometric conversion of substrate to product while also producing electrical current. This was shown by (Flynn et al. 2010) with an engineered *S. oneidensis* strain, which could convert glycerol to ethanol only in the presence of an oxidizing electrode. One of the many potential advantages of electrode-linked microbial catalysis is that it can be used as an electrochemical “lever” to drive an unfavorable reaction, enable the operation of a microbial fuel cell to generate electricity, or act as reducing equivalents for the synthesis of additional products (Rabaey and Rozendal 2010). (Bursac et al. 2017) developed a *S. oneidensis* strain which produced acetoin with a yield of 78% of the theoretical acetoin production maximum by electro-fermentation. Therefore, to increase the titers of the strains generated in this work, the next step would be to cultivate them in a BES with an oxidizing electrode. Future research will need to demonstrate whether it is possible to combine the two research fields - broadening the range of substrates and employing unbalanced fermentation - to achieve productivities that permit a competitive bio(electro)technological production using *S. oneidensis* as the producing strain.

In conclusion, our work effectively used metabolic engineering to boost *S. oneidensis’* production capacities. When compared to the wild type, the genetically engineered strain produced up to 25 mM glutamate, a remarkable 72-fold increase. Furthermore, for the first time, we showed that a genetically engineered *S. oneidensis* strain could produce itaconic acid. These findings support that it is possible to redirect carbon flux to the tricarboxylic acid (TCA) cycle in *S. oneidensis*. These findings enable future efforts to combine chemical production with electro-fermentation, a promising approach for sustainable and energy-efficient processes.

## Supplementary Information

Below is the link to the electronic supplementary material.

Supplementary file1 (PDF 117 KB)

## Declarations

### Data availability

All data generated or analyzed during this study are included in this published article and its supplementary information file. The supplementary information file contains Table S1 with all oligonucleotides, plasmids, and strains used in this work and Table S2 with retention times of analytical standards used for the quantification of metabolites by *S. oneidensis*.
