# Supplementary material for: Metabolic engineering of Shewanella oneidensis to produce glutamate and itaconic acid
Source: Appl Microbiol Biotechnol. 2024 Jan 6;108:36. doi: 10.1007/s00253-023-12879-5 (PMC10771365; doi:10.1007/s00253-023-12879-5)
Supplement: Supplementary file 1 [file 253_2023_12879_MOESM1_ESM.pdf]

Metabolic engineering of *Shewanella oneidensis* to produce glutamate and itaconic acid

Hannah Wohlers[1,2], Laura Zentgraf[2], Lisa van der Sande[1,3], Dirk Holtmann[3] *

1 Institute of Bioprocess Engineering and Pharmaceutical Technology, University of Applied Sciences Mittelhessen, Wiesenstrasse 14, 35390 Giessen, Germany

2 DECHEMA-Forschungsinstitut, Microbial Biotechnology, Theodor-Heuss-Allee 25, 60486 Frankfurt am Main, Germany

3 Institute of Process Engineering in Life Sciences, Karlsruhe Institute of Technology, Karlsruhe, Fritz-Haber-Weg 4, 76131 Karlsruhe, Germany

*Corresponding author,
Phone: + 49 (0) 721/608 - 421 31, e-mail address: dirk.holtmann@kit.edu

ORCID: D. Holtmann: 0000-0001-5540-3550

Supplementary tables

Table S1 Oligonucleotides, plasmids and strains used in this work (D. Hanahan and D.M. Glover 1985; S. G.N. Grant et al. 1990; Charles R. Myers and Kenneth H. Nealson 1988; Lassak et al. 2010; Jeske and Altenbuchner 2010).

Name	Genotype	Description/Application	Reference
**Bacterial strains**			
E. coli DH5α	F⁻ φ80*lac*ZΔM15 Δ(*lac*ZYA-*arg*F)U169 *rec*A1 *end*A1 *hsd*R17(r_K^-, m_K^+) *pho*A *sup*E44 λ⁻ *thi*-1 *gyr*A96 *rel*A1	Standard cloning applications	Hanahan (1985); Grant et al. (1990)
WM3064	*thrB1004 pro thi rpsL hsdS lacZ* ΔM15RP4–1360 Δ(*araBAD*)*567* Δ*dapA1341*::[*erm pir*(*wt*)]	Conjugation	W. Metcalf, University of Illinois
S. oneidensis MR-1	Wild type strain	Production strains	(Myers and Nealson 1988)
Δ2	*S. oneidensis* MR-1 with chromosomal deletion of Δ*ack* (SO2915) and Δ*pta* (SO2916)		This work
Δ*gltS*	*S. oneidensis* MR-1 with chromosomal deletion of Δ*gltS* (SO3562)		This work
Δ3	*S. oneidensis* MR-1 with chromosomal deletion of Δ*ack* (SO2915), Δ*pta* (SO2916) and Δ*gltS* (SO3562)		This work
**Plasmids**			
pNPTS138-R6KT	mobRP4 + ori-R6K, sacB; suicide plas-<br>mid for in-frame deletions; *KanR*	Suicide plasmid for in-frame deletions	Lassak et al 2010
pNPTS138-R6KT_ackApta	500 bp upstream region of *ackA* and 500 bp downstream region of *pta* in pNPTS138-R6KT		This work
pNPTS138-R6KT_gltS	500 bp upstream and downstream region of *gltS* in pNPTS138-R6KT		This work
pJeM1	*rhaR rhaS rhaPBAD eGFP*, mob, *KanR*	Expression vector for fluorescence assay	Jeske und Altenbucher 2010
pG2	Based on pJeM1, *rhaR rhaS*, *rhaPBAD gdhA* (GenBank accession no. CP025534.1), *NCgl1221 A111V* (GenBank accession no. CP025534.1), Mob	Expression vector for glutamate production	This work
pIA	Based on pJeM1, *rhaR rhaS, rhaPBAD cadA* (GenBank accession no. MH366503.1), *acnB* (GenBank accession no. AE014299.2), Mob	Expression vector for itaconic acid production	This work
**Oligonucleotides**			
pNPTS138_fw	ccgaagctagcgaattcgtgg	Amplification of pNPTS138-R6KT	This work
pNPTS138_rev	gtacaggcatgcgtcgacc		

		backbone for Gibson assembly	
OL_up_gltS_down_fw	gagtctagtgatgtacccacacaaaccatttgtaaggaactatcccaaacaataataaaaatggcgccta	Amplification of up- and downstream fragments	This work
gltS_down_OL_pNTPS_rv	ctcacttaaggccttgactagagggtcgacgcatgcctgtacgtggggatattgtcgtatttgactc		
OL_pNTPS138S_ackA_fw	ccaagcttctctgcaggatatctggatccacgaattcgctagcttcgggcccagggattggcaatgtatg		
Pta_OL_pNTPS138_rev	cacttaaggccttgactagagggtcgacgcatgcctgtacaacaaaaacagtaatgaaagaaaaaat		
Seq_ackA_fw	taacgacgaccatcacc	Verification of gene deletions	This work
Seq_pta_rev	gcgtttaatgctgtcacat		
Seq_gltS_fw	cgaacaggcgatgtaggc		
Seq_gltS_rev	cggataaaaggctcattaag		
pJeM1_fw	atgtatatctccttcttaagaattgttc	Amplification of pJeM1 backbone for Gibson assembly	This work
pJeM1_rev	gcttaccggtttattgactacc		
pJeM1_OL_GDH2_fw	actggtcgtaatgaacaattcttaagaaggagatatacatatgacagttgatgagcaggt	Amplification of *gdhA* fragment	
GDH2_Link_RBS_rev	atgtatatctccttcttaagaattgaccaccaccagcttagatgacgccctgtgc		
Link_RBS_OL_NCgl1221_fw	atctaagctggtggtggtcaattcttaagaaggagatatacatatgattttaggcgtacccattc	Amplification of *NCgl1221 A111V* fragment	
NCgl1221_OL_pJeM1_rev	cacggtcacactgcttccggtagtcaataaaccggtaagcctaaggggtggacgtcg		
Seq1_fw	cattttcctgtcagtaacgaga	Verification of pG2	This work
Seq2_fw	aagggcgagagcatcagc		
Seq3_fw	aggcgtacccattcaatatttgc		
Seq4_fw	ggtgatcaatgcaggcaatcc		
Seq5_rev	cgtcaattattacctccacgg		
Seq6_rev	atgtatatctccttcttaagaattg		
pJeM1_oGFP_fwd	cgtaatcatggtcatatgtatatc	Amplification of pJeM1 backbone for Gibson assembly	This work
pJeM1_oGFP_rev	gcttaccggtttattgac		
cadA_acnB_fwd_pJeM1PCR	tagtcaataaaccggtaagcttatacagctgattgaaagatc	Amplification of *cadA* and *acnB* fragment for Gibson Assembly	This work
cadA_acnB_rev_pJeM1PCR	tacatatgaccatgattacgatggctaaacaatctgctg		
cadA_Seq1	ctggtcgtaatgaacaattc	Verification of pIA	This work
cadA_Seq2	gatgattaccactctgaagc		
cadA_Seq3	gctcgtaacggtttattagg		
cadA_Seq4	tgcttacatcttagctgttc		
cadA_Seq5	aactgtccagttaaatctcc		

Table S2 Analytical standards and retention times used for the quantification of metabolites by *S. oneidensis*.

**Standard substance**	**Retention time [min] HPLC**	**Retention time [min] LC-MS/MS**
Lactate	12.48	n.d.
Itaconic acid	15.30	n.d.
Acetate	14.77	n.d.
Glutamate	n.d.	0.78

Supplementary references

Charles R. Myers, Kenneth H. Nealson (1988) Bacterial manganese reduction and growth with manganese oxide as the sole electron acceptor. Science 240:1319–1321. doi: 10.1126/science.240.4857.1319

D. Hanahan, D.M. Glover (1985) Techniques for transformation of *E. coli*. DNA cloning: a practical approach. Appl Environ Microbiol 65(12): 5303–5306. doi: 10.1128/aem.65.12.5303-5306.1999

Jeske M, Altenbuchner J (2010) The *Escherichia coli* rhamnose promoter *rhaP(BAD)* is in *Pseudomonas putida* KT2440 independent of Crp-cAMP activation. Appl Microbiol Biotechnol 85:1923–1933. doi: 10.1007/s00253-009-2245-8

Lassak J, Henche AL, Binnenkade L, Thormann KM (2010) ArcS, the cognate sensor kinase in an atypical arc system of *Shewanella oneidensis* MR-1. Appl Environ Microbiol 76:3263–3274. doi: 10.1128/AEM.00512-10

S. G.N. Grant, J. Jessee, F. R. Bloom, D. Hanahan (1990) Differential plasmid rescue from transgenic mouse DNAs into *Escherichia coli* methylation-restriction mutants. PNAS 87:4645–4649. doi: 10.1073/PNAS.87.12.4645
